# Rhythmic Ganglion Cell Activity in Bleached and Blind Adult Mouse Retinas

**DOI:** 10.1371/journal.pone.0106047

**Published:** 2014-08-25

**Authors:** Jacob Menzler, Lakshmi Channappa, Guenther Zeck

**Affiliations:** 1 Neurochip Research Group, Natural and Medical Sciences Institute at the University Tübingen, Reutlingen, Germany; 2 Helmholtz Zentrum München, Deutsches Forschungszentrum für Gesundheit und Umwelt, Neuherberg, Germany; Instituto Murciano de Investigación Biosanitaria-Virgen de la Arrixaca, Spain

## Abstract

In retinitis pigmentosa – a degenerative disease which often leads to incurable blindness- the loss of photoreceptors deprives the retina from a continuous excitatory input, the so-called dark current. In rodent models of this disease this deprivation leads to oscillatory electrical activity in the remaining circuitry, which is reflected in the rhythmic spiking of retinal ganglion cells (RGCs). It remained unclear, however, if the rhythmic RGC activity is attributed to circuit alterations occurring during photoreceptor degeneration or if rhythmic activity is an intrinsic property of healthy retinal circuitry which is masked by the photoreceptor’s dark current. Here we tested these hypotheses by inducing and analysing oscillatory activity in adult healthy (C57/Bl6) and blind mouse retinas (*rd10* and *rd1*). Rhythmic RGC activity in healthy retinas was detected upon partial photoreceptor bleaching using an extracellular high-density multi-transistor-array. The mean fundamental spiking frequency in bleached retinas was 4.3 Hz; close to the RGC rhythm detected in blind *rd10* mouse retinas (6.5 Hz). Crosscorrelation analysis of neighbouring *wild-type* and *rd10* RGCs (separation distance <200 µm) reveals synchrony among homologous RGC types and a constant phase shift (∼70 msec) among heterologous cell types (ON versus OFF). The rhythmic RGC spiking in these retinas is driven by a network of presynaptic neurons. The inhibition of glutamatergic ganglion cell input or the inhibition of gap junctional coupling abolished the rhythmic pattern. In *rd10* and *rd1* retinas the presynaptic network leads to local field potentials, whereas in bleached retinas additional pharmacological disinhibition is required to achieve detectable field potentials. Our results demonstrate that photoreceptor bleaching unmasks oscillatory activity in healthy retinas which shares many features with the functional phenotype detected in *rd10* retinas. The quantitative physiological differences advance the understanding of the degeneration process and may guide future rescue strategies.

## Introduction

A major excitatory current in the retina is continuously generated by photoreceptors in the dark. This so-called dark-current increases in the developing retina, maintains a constant level throughout adulthood [1] and eventually disappears in the degenerative disease of retinitis pigmentosa, where mutations of the PDE6β [2] gene leads to rod degeneration and ultimately to incurable blindness.

The rod degeneration may be accompanied by functional alterations of the electrical activity of inner retinal neurons. It has been reported recently that the projection neurons in dystrophic mouse retinas exhibit hyperactivity [3], [4] which is rhythmic in many of the detected retinal neurons [5], [6], [7], [8], [9]. The origin of the rhythmic ganglion cell spiking in *rd1* retinas – a mouse model of retinitis pigmentosa [2] - was assigned to presynaptic input [5], [6], [7], [8]. It may originate in the electrically coupled ON cone bipolar –amacrine cell network [10] or the AII amacrine cell alone [11] and effect the OFF pathway through chemical synapses [9] Rhythmic ganglion cell activity with lower fundamental frequency (∼5 Hz) compared to *rd1* has been reported for ganglion cells of the *rd10* mouse [12] – an rd model [2] where photoreceptor degeneration occurs later in development. Ganglion cells in other retinal disease models develop a rhythmic (∼5 Hz) activity as well, i.e. in Leber congenital disease where photoreceptors fail to fully develop [13] or in congenital stationary night blindness (n*ob*-mouse [14]. A second characteristic electrophysiological feature found in *rd1* retinas are spatially extended changes of the extracellular potential detected in the ganglion cell layer [7]. These local field potentials indicate large-scale depolarisations originating from concerted presynaptic activity.

It remained unclear, if the rhythmic RGC spiking and emergence of local field potentials in *rd1* were caused by changes in retinal circuitry or if they are intrinsic properties of retinal circuitry, which are masked in the healthy retina. A recent study [15] reports wave-like propagating activity in pharmacologically disinhibited retinas; however at frequencies smaller 1 Hz.

Here, we test to what extent partial photoreceptor bleaching in *ex* *vivo* healthy retinas induces physiological activity that resembles the *rd* phenotype. This experiment is motivated by the so-called ‘equivalent light hypothesis’ [16] which postulates that the loss of photoreceptors is equivalent to the situation in which the rods are continuously hyperpolarized, as they would be during saturating continuous light.

Extracellular recording of RGC activity was performed in adult wild-type (C57/Bl6) and in two adult mouse models of retinal degeneration (*rd10* and *rd1*). We used a high- density CMOS based micro-electrode arrays (7.4 µm spatial resolution) which allow for precise mapping (‘electrical imaging’) of single cell activity as well as the spatial mapping of local field potentials [17].

Our results demonstrate that rhythmic ganglion cell activity with similar statistics is recorded in healthy and blind retina. However, we also note differences regarding the emergence of local field potentials and synchronicity which can only be mimicked in healthy retinas by lowering inhibition through pharmacological blockers.

## Materials and Methods

### Animals

All procedures were approved by the animal use committee of the Natural and Medical Science Institute at the University Tübingen (Approval Number NMI 33) and performed in compliance with the ARVO statement for the use of animals in Ophthalmic and Visual Research. Protocols compliant with § 4 paragraph 3 of the German law on animal protection were reviewed and approved by the “Einrichtung für Tierschutz, Tierärztlichen Dienst und Labortierkunde” (Anzeige/Mitteilung nach § 4 vom 29.10.2012). All efforts were made to minimize the number of animals used and their suffering.

In this study adult male C57BL/6N mice, B6.CXB1-Pde6brd10/J (*rd10)* and C3H/HeJ (*rd1)* mice, all between postnatal day P90–P100 were used. All animals were housed in temperature regulated facilities on a 12 h light/dark cycle and fed *ad libitum*. Different background strains in *rd1* (C3H and C57BL) do not lead to different physiological results [3]. We therefore compare the previously [6] used *rd1* (C3H) strains to rd10 (C57BL). All animals were light adapted 2 hours before the retina preparation. In some cases the preparation was performed under dim red light [7]. These retinas were used to investigate if the pharmacological block alone can induce rhythmic activity. All other retinas were prepared in ambient light.

### Recording

The extracellular electrical activity of the retina was measured using a high-density CMOS microelectrode array comprising 128×128 equally spaced recording sensors which cover an area of 1 mm^2^. In this study, we measured every second column (128×64 sensors) with a sampling frequency of ∼10 kHz for each sensor. Details of the recording technique are described in [7], [17]. During the recording, the retina was continuously superperfused with carbogenated Ames’ medium (A 1420, Sigma).

#### Identification of action potentials and assignment to the corresponding ganglion cell

The identification of an action potential on the high-density multi-transistor-array is performed offline in two steps as described in detail in (Lambacher et al. 2011; Menzler&Zeck, 2011). In the first step threshold crossings of a signal *V* are identified. The signal *V* is calculated from extracellular voltages recorded on neighbouring sensors (3×3 environment) and by considering 3 consecutive timesteps 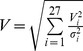, with 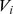 representing the signal amplitude of measured data point *i*, 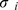: root mean square (*rms)* noise of the corresponding sensor. The sum runs over the 3×3×3 neighbourhood surrounding the data point under consideration. If *V* exceeds a threshold of 15 the data point is saved and considered part of the extracellular action potential waveform. In the second step threshold crossings were combined to one action potential (Menzler and Zeck 2011). The data point (time stamp and sensor location) with the highest amplitude is chosen as a representative for the action potential.

#### Assignment of an action potential to the corresponding ganglion cell

The ganglion cell layer of mammalian retinas contains the somata of ganglion cells and of putative spiking amacrine cells. To prove that activity is indeed recorded from ganglion cells we electrically imaged the signal propagation along the proximal axon [18]. For the action potentials of one given neuron a so-called average electrical image is calculated. The spike triggered average electrical image is obtained by averaging the extracellular voltages recorded by each of the 8192 sensors in a defined time window around the threshold crossings for at least thirty action potentials. For each cell we obtain a characteristic “footprint” of sensors on the array. To properly assign the spikes to a given ganglion cell we selected sensors which are part of only footprint.

#### Data analysis

Maintained RGC activity was calculated after concatenation of about one hundred subsequent continuous recording segments (∼1 second each). We confirmed that overall maintained activity did not vary if different recording intervals are selected. Spike trains assigned to the corresponding cells were binned with either 4 or 0.4 ms time resolution. Normalized cross-correlation (CC) functions of these histograms were computed using Matlab (The MathWorks).

Light-evoked RGC responses were recorded upon presentation of a large spot (1 mm diameter) for 500 msec. Ganglion cells which respond to light onset were assigned to the ON subclass, cells that respond to light offset only were assigned to OFF cells. ON–OFF ganglion cells are not considered because of the relatively small number of identified cells using the ‘full-field’ stimulus. Transient and sustained cell types are pooled within the given cell class (ON and OFF). To test for statistical significance of firing rate or fundamental frequency, the Wilcoxon-Mann-Whitney *U* test was used. Average values are given as mean ± standard error of the mean.

#### Bleaching

Spontaneous RGC activity was recorded for more than three hours after the retina had been interfaced to the sensor array. For the first thirty minutes the retinal activity was recorded in darkness, and then light responses were measured for about 20 minutes. Afterwards the retina was bleached for 2 hours by full-field white light. The light was presented on a miniature monochrome organic light emitting diode display (oLED; eMagin Corp., Bellevue, WA) illuminating the back focal plane of a 5x objective (LMPlan Fl; Olympus Optical, Tokyo, Japan). The light intensity (40–80 mW/m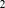) in the focus plane (location of the interfaced retina) was measured using an optical meter (1835-C, Newport Spectra-Physics, Darmstadt, Germany). The emission spectrum of the oLED monitor ranges between 430–700 nm. The monitor thus bleaches rods and m-cones. The mouse s-cones, however, are not affected by this oLED spectrum. The procedure applied here is thus considered a “partial” bleaching.

#### Pharmacology

To block glutamate-sensitive AMPA/kainate receptors, 6,7-Dinitroquinoxaline-2,3-dione disodium salt (DNQX, 20 µM) was used. Glycinergic receptors were blocked by strychnine (2 µM), while for GABA_A_ receptors SR-95531 hydrobromide (gabazine, 20 µM) was used. All antagonists were purchased from Tocris. Gap junctions were blocked by Meclofenamic acid (100 µM, Sigma). The antagonists were dissolved in Ames’ solution and perfused to the interfaced retinal tissue.

## Results

### Identification of RGCs in the mouse retina

**Figure 1:**
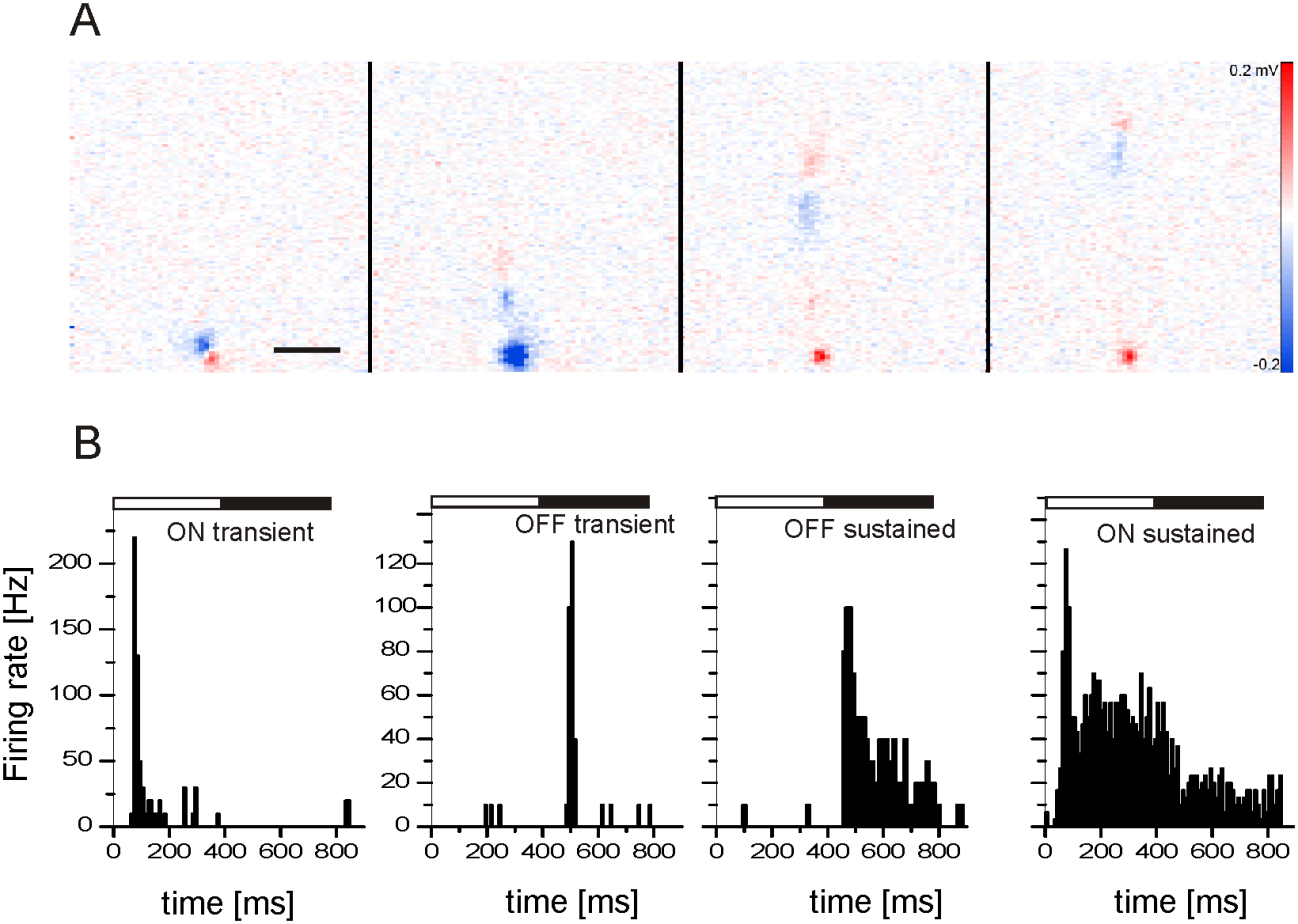
Identification of RGCs using the high-density multi transistor array. (A) Four consecutive electrical images acquired every 100 µsec reveal the occurrence and propagation of action potentials along the proximal axon. The electrical images were obtained after calculating the spike triggered average over more than thirty spikes. Visualization of the axon identifies a ganglion cell (Methods). Scale bar: 200 µm. (B) Typical light responses of four RGCs in wild-type retinas. The histograms were calculated for repeated stimulus presentations. (binsize: 5 ms).

To identify retinal ganglion cells in extracellular recordings we calculated a mean electrical image as a spike triggered average (STA) map of the sensor array (Methods). The STAs reveal the extracellular signal underneath the RGC soma and additionally the propagating action potential along the unmyelinated axon (**Fig. 1A**) [18], [19]. STAs comprising proximal axon pathways unambiguously separate RGCs from putative spiking amacrine cells in the RGC layer. The identified RGCs were classified upon their light response into ON- and OFF-RGCs (**Fig. 1B**). Sustained and transient cell types were not considered separately, mainly because a restricted stimulus set was presented which did not allow for rigorous cell classification.

### RGC spiking in constantly illuminated C57/Bl6 retinas is similar to spiking in rd10 retinas

In the first experiment we investigate the population activity of retinal ganglion cells (RGC) in a healthy retina during constant illumination. This illumination leads to a decrease of the photoreceptor dark current. The reduction or disappearance of glutamate release may be in a certain way equivalent to the degeneration of photoreceptors [16].

**Figure 2:**
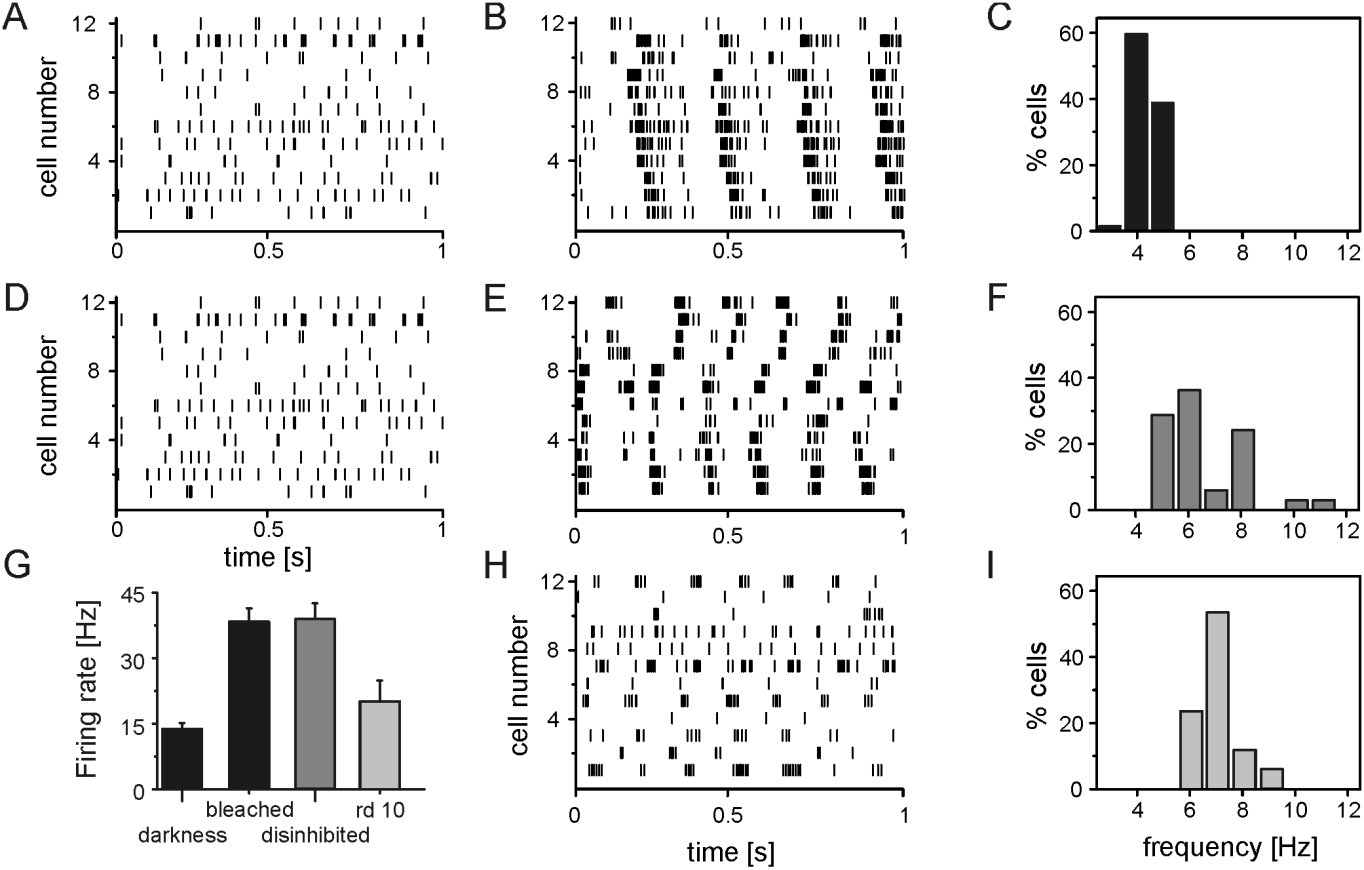
RGC spike train properties in bleached, disinhibited and *rd10* retinas. A) Rasterplot of spontaneous RGC activity recorded in C57BL/6 retinas in darkness. Each tick represents one action potential. B) Activity recorded from the same cells shown in (a) after 2 hrs of illumination. C) Histogram of the fundamental frequency of 65 rhythmic RGCs measured in three bleached retinas. D) Rasterplot of spontaneous RGC activity recorded in C57BL/6 RGCs in darkness before disinhibition. E) Activity recorded from the same cells shown in (D) after exposure to light and the application of inhibitory receptor blockers (strychnine and gabazine). F) Histogram of the fundamental frequency of 111 RGCs measured in four disinhibited retinas. G) Average RGC maintained activity measured in *wt* retinas in darkness, in bleached condition and in disinhibited condition and average maintained activity of *rd10* RGCs. H) Rasterplot of spontaneous RGC activity recorded from *rd10* retinas. I) Histogram of the fundamental frequency of 32 rhythmic RGCs evaluated from three *rd10* retinas.

We analyzed the activity of 126 identified RGCs in three partially bleached retinas. First we evaluated the firing rate in darkness (**Fig. 2A**) and found a mean value of 13 Hz±1.3 Hz, mean + s.e.m). This value is in the same range of previously reported firing rates [6], [7]. Maintained firing rates were similar for the ON- (n = 86 cells) and OFF-RGC (n = 40 cells) subpopulations. Constant illumination of the retinas (see Material and Methods) increased the average firing rate shortly after onset to 38 Hz±3.6. After a long interval (>2 hr) of constant illumination, rhythmic spiking was observed in 65 RGCs (52% of the total population). The raster plots of twelve rhythmic RGCs recorded in the same retinas is shown in **Fig. 2B**. The presented cells were of both types (ON and OFF). The autocorrelations of the rhythmic spike trains displayed multiple peaks, with the first peak occurring at ∼250 ms. This value translates to a fundamental frequency of the ganglion cell rhythm of 4 Hz. The average fundamental frequency calculated for all rhythmic RGCs was 4.3±0.1 Hz. The distribution of fundamental frequencies is shown in **Fig. 2C**. Both major cell classes (ON and OFF RGCs) displayed rhythmic activity (28 ON and 30 OFF RGCs, 7 unidentified RGCs). We like to emphasize, that bleaching is only partial in our experiments. When we stimulate the retina after the long constant illumination using a 1 Hz flicker stimulus, the light responsiveness of RGCs recovers after several minutes (Figure S1), in line with a recent report (A. Tikidji-Hamburyan, IOVS 2014: ARVO e-Abstract 5961).

Is the RGC hyperactivity caused by an increase of the excitatory driving force or by a decrease of inhibition? To answer this question we blocked the inhibitory receptors during the partial bleaching of wild-type retinas (Material and Methods). We analyzed 153 RGCs (4 retinas, 79 ON-cells; 74 OFF-cells). The average maintained firing rate increased from from 12±2 Hz in darkness to 39 Hz±2.5 Hz during constant illumination. Disinhibition of the retina by the blocker mixture Strychnine and gabazine 20 minutes after the onset of illumination led to oscillatory spiking in 111 RGCs (72%). 43 of the rhythmic RCS were of ON- and 71 OFF RGCs. Inhibitory blockers changed only the spiking pattern but not the firing rate. The mean fundamental frequency of rhythmic RGCs was 6.5±0.3 Hz; however there was a large variability across cells (**Fig. 2F**). Application of either Strychnine or gabazine alone did not lead to rhythmic firing. After wash out of the blocker mixture the rhythmic spiking disappeared. We conclude that inhibition of the dark current or blockade of synaptic inhibition generates a phenotype with rhythmic bursting RGCs.

To compare the induced rhythmic activity with a model of retinal degeneration in detail, we recorded RGC activity from adult *rd10* retinas (n = 3, P90–P100), when all photoreceptors are lost (Gargini et al., 2007). The mean firing rate calculated from 62 RGCs was lower (20±2 Hz) than in constantly illuminated retinas (**Fig. 2G**) and lower compared to *rd1* RGCs (26±13 Hz, [7]) RGCs of the same age. On the other hand the proportion of rhythmic firing cells was 50%, which is similar to bleached retinas. This percentage is however; lower than the percentage of rhythmic RGCs in disinhibited retinas or in *rd1* retinas under otherwise identical conditions. The mean fundamental frequency evaluated from 32 rhythmic *rd10* RGCs was 6.5±0.2 Hz (**Fig. 2I**). This value is significantly higher than the average fundamental frequency of bleached retinas (p = 0.001) but not significantly different from the fundamental frequencies recorded in disinhibited retinas (p = 0.39).

In summary, in bleached as well as in *rd* retinas qualitatively similar rhythmic spiking is detected. Quantitatively, the values for maintained activity or rhythmicity (fundamental frequency) differ. In the following we evaluate the pair-wise correlation between RGCs, which helps identifying the driving force of rhythmic activity.

### Similarities of pair-wise spike correlation properties in bleached and *rd10* retinas

**Figure 3:**
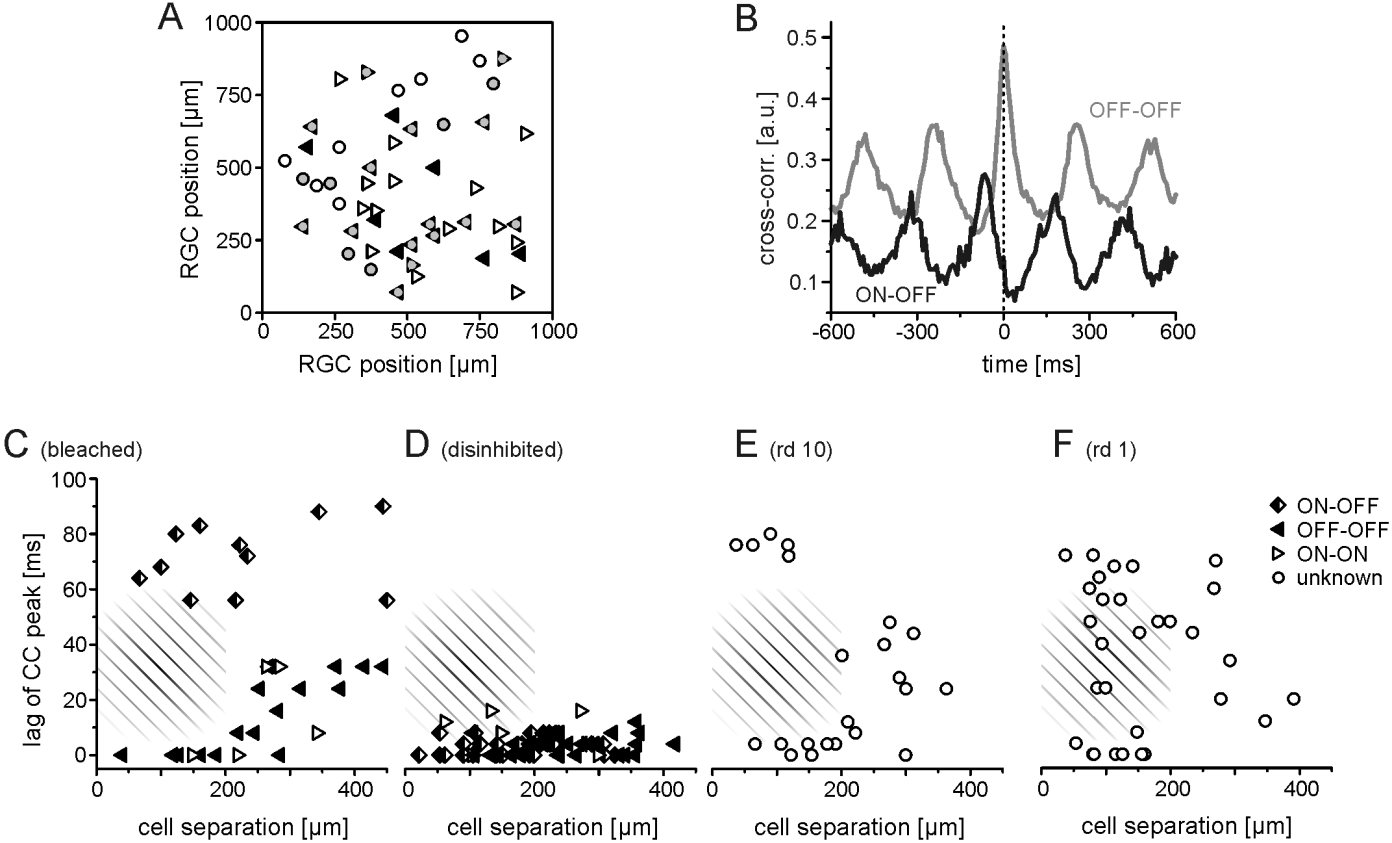
Pair-wise spike correlation properties in bleached and dystrophic retinas. (A) Spatial locations of 53 identified RGCs in one bleached C57/Bl6 retina. Symbols denote OFF RGCs (filled triangle), ON RGCs (open triangles), and physiologically unidentified RGCs (open circles). The rhythmic RGCs are marked with filled grey circles. (B) Cross-correlogramm of the spike trains recorded from two rhythmic OFF RGCs (gray) and a pair of one rhythmic ON and a rhythmic OFF RGCs (black). The OFF-OFF pair fires in synchrony (zero time lag), while RGCs of different polarity display a phase shift. The time lag of the central peak is marked with a dashed line. The rhythmic activity is reflected in the auxiliary peaks of the CC. (C) Dependency of the CC time lag shift for RGC pairs and the cell separation. The legend for the three cell type combinations is given in subplot (F). The hatched area illustrates that RGC pairs separated by less than 200 µm do not display arbitrary lags. This area is drawn for comparison in the following subplots as well. (D–F) Time shift of the central peak in the CCs computed for rhythmic RGC pairs in disinhibited retinas (D), *rd10* retinas (E) and rd1 retinas (F). The open symbol denotes unknown RGC cell type.

Rhythmic ganglion cells fire either in synchrony or phase shifted. To estimate the temporal relation between pairs of RGCs the cross-correlogram (CC) of their spike trains are calculated (**Fig. 3A**). For retinal ganglion cells in non-rhythmic *wt* retinas nearby cells have been reported to fire in synchrony – either mediated by gap junctional coupling or driven by a common presynaptic interneuron [20]. To distinguish between the two possibilities the CC is computed at high temporal resolution. Gap junctional coupling results in a double-peaked CC whereas a common presynaptic driving force leads to one central peak without any time lag. For the spike trains analyzed here very few RGC pairs displayed gap-junctional coupling, which was always superimposed by presynaptic input.

We performed the cross-correlation analysis for all RGCs which showed clear light responses. The RGCs in one recorded portion were classified as rhythmic or non-rhythmic, based on the equidistant peaks in the spike autocorrelograms. The locations of the identified RGC somata in a bleached retina are shown in **Fig. 3A**. Remarkably, rhythmic and non-rhythmic RGC are found at random positions, i.e. there is no partition in bleached retinas which contains only rhythmic RGCs. We performed the cross-correlation analysis separately for rhythmic cells of the same polarity (ON-ON and OFF-OFF) and for cells of different polarity (ON-OFF) in bleached wt retinas. Two example CCs are shown in **Fig. 3B**.

For cell separation distances below 200 µm and homologous RGC pairs (ON-ON or OFF-OFF) the time shifts of the central peak were always <10 msec. For soma separations larger than 200 µm the time shift takes arbitrary values. RGCs of different polarity exhibited a shift of ∼70 msec if their cell bodies were separated by less than 200 µm and arbitrary values for larger time shifts (**Fig. 3C**). For neighboring pairs of ON and OFF RGCs the ON cell activity always precluded the OFF cell spiking. This result suggests that nearby cells of the same polarity are driven by the same presynaptic cell(s) while the shifted rhythm for ON-and OFF ganglion cells is mediated by a small interneuron (∼200 µm), which introduces the observed shift through an additional synapse.

We repeated the analysis for the rhythmic RGCs recorded in disinhibited retinal preparations (**Fig. 3D**). In these retinas most rhythmic RGCs fire in synchrony even over distances longer than 200 µm (**Fig. 3D**). Synchronous spiking was detected for heterelogous cell pairs, in contrast to the findings for bleached retinas. The identification of ON and OFF RGCs was performed before the addition of inhibitory blockers. A few RGCs were still out of phase suggesting that some inhibitory receptors remain functional.

How does the rhythmicity in dystrophic retinas compare to that recorded in bleached retinas? For RGCs pairs in *rd10* retinas we observed the same behavior as seen in bleached retinas (compare **Fig. 3C** and **Fig. 3E**). For cell separation distances smaller than 200 µm the spike trains exhibit either no phase shift or a phase shift of ∼70 msec. In these blind retinas we were not able to establish the cell polarity (ON or OFF RGC). The forth investigated retinal preparation stems from adult blind *rd1* retinas. The cross-correlation analysis in these retinas reveals very different results compared to the *rd10* RGCs (**Fig. 3F**). The peak time-lags do not spare the hatched area, indicating that there was no distinct phase shift for any RGC separation distance. This results will be discussed below.

We conclude that based on the analysis of pair-wise cross-correlograms the RGCs in bleached *wt* retinas and in *rd10* retinas display similarities which are qualitatively different from disinhibited light-exposed *wt* retinas or *rd1* retinas respectively. In the following we test if the rhythmic RGC activity in bleached or rd10 RGCs has a presynaptic origin.

### Rhythmic RGC activity in bleached C57/Bl6 and rd10 retinas has a presynaptic origin

**Figure 4:**
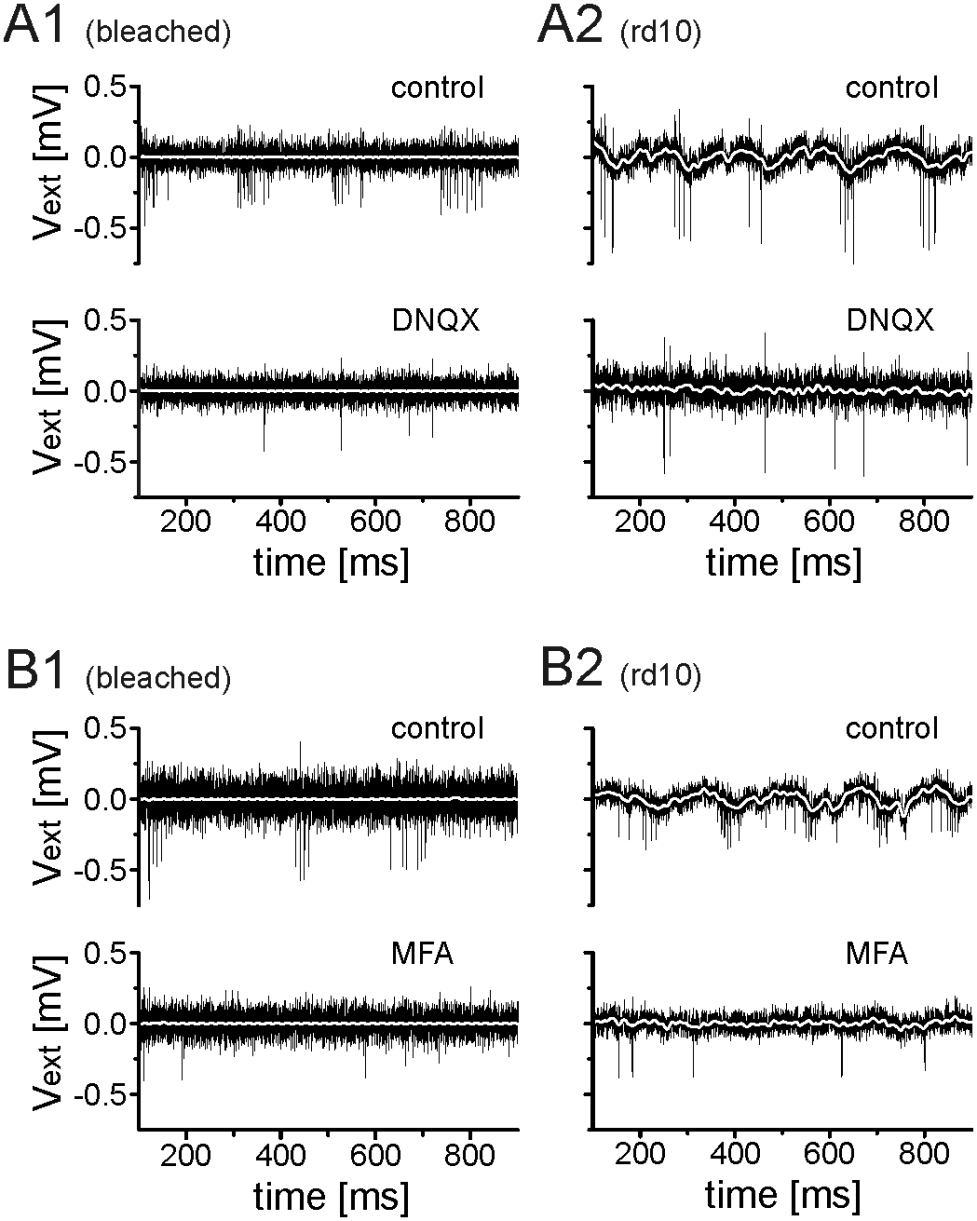
Rhythmic RGC spiking is abolished by ionotrophic glutamate receptor blocker or by gap junction blockers in bleached and in *rd10* retinas. (A1 & A2) DNQX (20 µM) prevents rhythmic RGC activity in bleached retinas (A1) and in rd10 retinas (A2). (B) The gap junction blocker MFA (100 µM) inhibits rhythmic spiking in bleached (B1) and in *rd10* retinas (B2). White lines mark the 100 Hz low-pass signal for each trace.

It has been reported that the network of electrically coupled ON bipolar cells and amacrine cells display ∼5 Hz rhythmic activities in dystrophic *rd1* retinas [8], [10]. This network drives the rhythmic spiking in *rd1* RGCs. Here we investigated if the presynaptic circuitry is responsible for rhythmic RGC spiking in bleached and *rd10* retinas as well. We therefore blocked the major ionotrophic glutamatergic receptors of RGCs using the AMPA/kainite receptor blocker DNQX (20 µM). In both retinas (bleached and *rd10*) the rhythmic ganglion cell spiking disappeared (**Fig. 4A** **1** and **Fig. 4A** **2**).

Next, we assessed if electrical coupling is necessary for the rhythmic activity in these retinas. The rhythmic activity disappeared in bleached C57Bl6 and *rd10* retinas after the addition of 100 µM MFA (**Fig. 4B** **1** and **Fig. 4B** **2**). This result suggests that the previously described network of electrically coupled bipolar and amacrine cells [10] is likely responsible for the rhythmic activity in these retinas as well. We note, that the application of a smaller concentration of MFA (10 µM) did not lead to the disruption of rhythmic RGC activity. Possible side effects of MFA are critically reviewed in the discussion section.

The rhythmic spiking in bleached and rd10 retinas differs in the low-frequency range. In the following we investigate if the low frequency oscillations detected in *rd10* represent spatially extended network depolarizations and how these compare to bleached *wt* or to disinhibited retinas.

#### Local field potentials differ in C57/Bl6 and dystrophic retinas

Local field potentials (LFPs) reflect changes in the low-frequency range (<100 Hz) of the extracellular voltage measured between the sensor array and the ground electrode. The spontaneous emergence [7], [21] and propagation of LFPs [7] constitutes a characteristic feature of blind *rd1* retinas. In untreated or in bleached *wt* retinas we could not detect any low-frequency changes of the extracellular voltage. As the continuous recording of the entire sensor array was restricted to a few seconds in this study, the minimal frequency set by the Nyquist sampling-theorem we can detect is ∼1 Hz.

**Figure 5:**
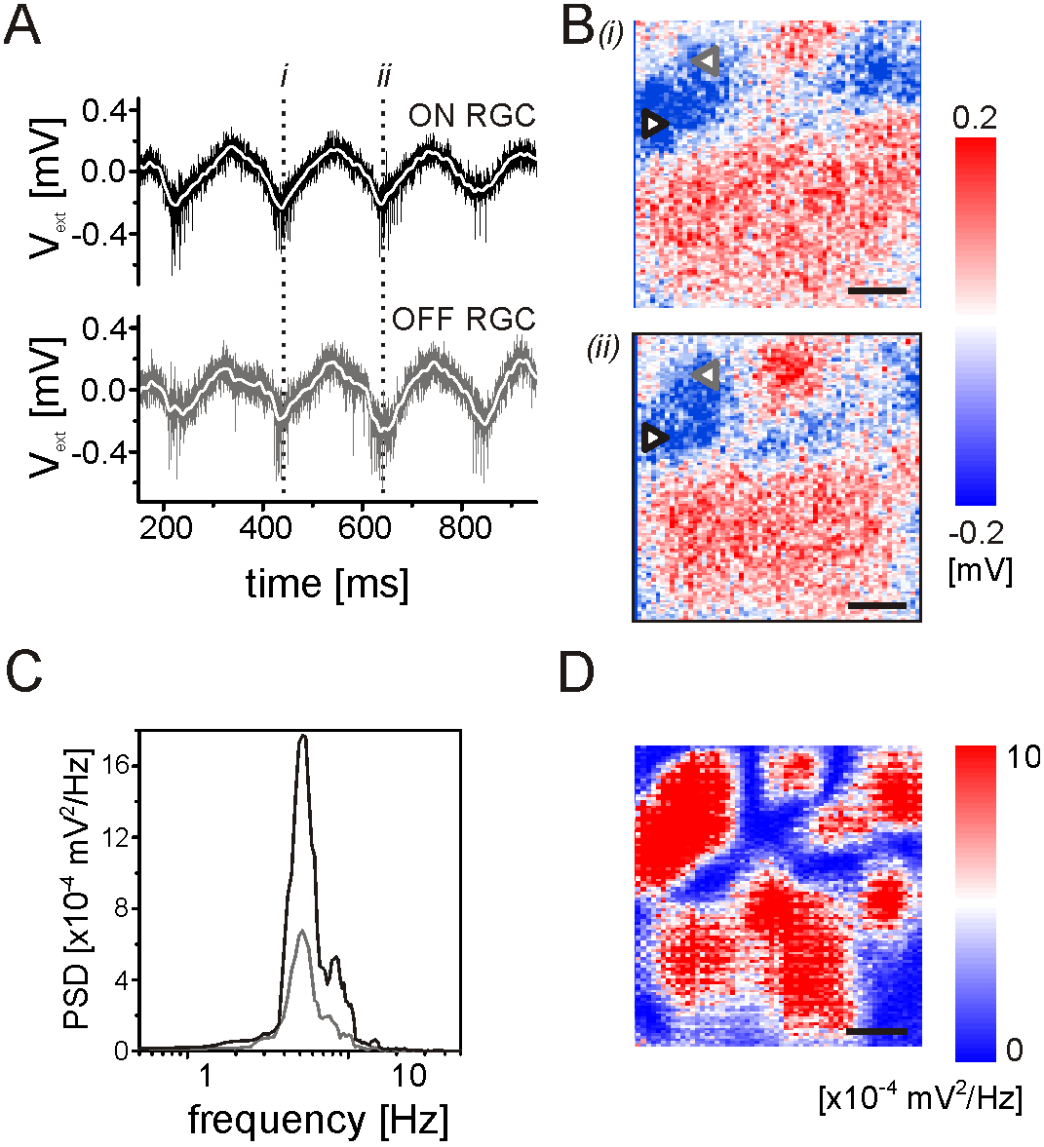
Rhythmic RGC spiking and strong stationary LFPs emerge during application of glycinergic and GABAergic receptor blockers in bleached mouse retinas. A) Extracellular voltage traces recorded on two different sensors on the multi-transistor-array. The upper trace shows activity recorded from an ON RGC, the lower trace shows activity recorded from an OFF RGC. (B) Local field potentials recorded on the whole sensor array at the two time points marked in (A). The symbols mark the position of the sensors shown in (A). C) Power spectral density evaluated for the two sensors reveals a maximum at the frequency of ∼6 Hz. (D) The amplitude of the PSD (as shown in C) measured at all sensors at the maximal frequency. Scale bars in (B) and (D): 200 µm.

LFPs were detected in disinhibited *wt* retinas after perfusion with the blocker mix of strychnine and gabazine. Rhythmic RGC spiking (**Fig. 5A**) was accompanied by spatially extended LFPs as illustrated in **Fig. 5B**. The LFPs covered areas that were often larger than the recording array (1 mm^2^, **Fig. 5B**). We were not able to identify any propagation of the LFPs as detected previously in *rd1* retinas [7]. Addition of ionotrophic glutamate receptor blocker (DNQX) to the inhibitory blocker mix abolished the LFPs and also the rhythmic RGC spiking. The amplitude of the LFPs was assessed by power spectral analysis. The maximal LFP amplitude varied between 2×10^−4^–10^−3^ mV^2^/Hz on the different sensors (two of them are shown in **Fig. 5C**). The representation of the LFP amplitude on all recording sensors evaluated at the fundamental frequency is shown in **Fig. 5D**. This representation allows estimating the spatial LFP extent throughout the recording.

**Figure 6:**
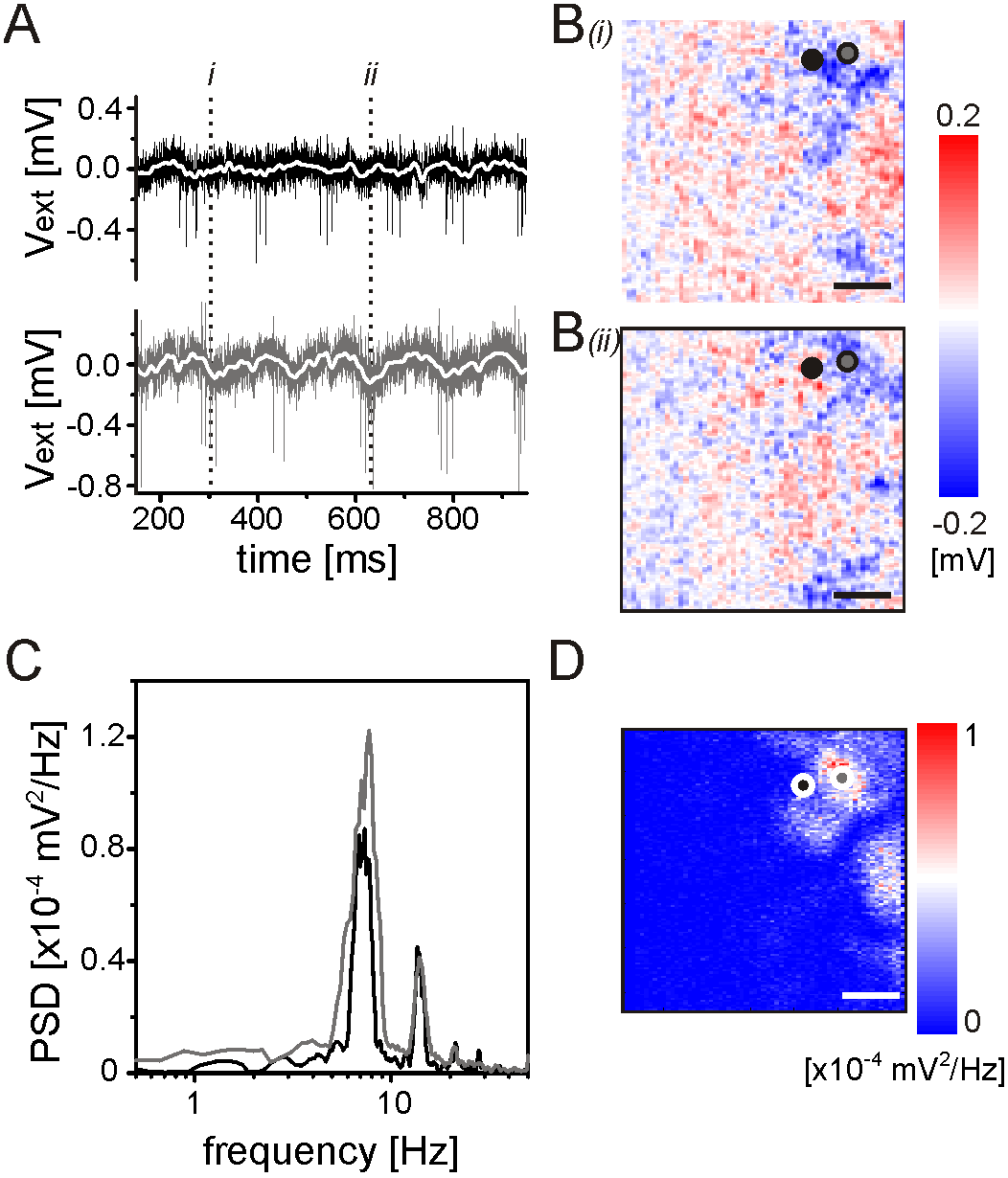
Rhythmic spiking and weak stationary LFPs in the ganglion cell layer of adult *rd10* mouse retinas. A) Calibrated voltage traces recorded on two selected sensors. The sensor positions are marked in panel (B). The rhythmic spiking of the two RGCs is not in phase. B) Local field potentials measured at the time points marked in (A). (C) Power spectral density evaluated at the two sensors reveals the fundamental rhythm at ∼7 Hz. (D) The amplitude of the PSD (as shown in C) measured at all sensors at the maximal frequency. Scale bars in (B) and (D): 200 µm.

In the ganglion cell layer of blind *rd10* retinas we recorded rhythmic RGC activity which is driven by LFPs as well (**Fig. 6A**). The LFPs detected did not propagate in the RGC layer of *rd10* retinas either. Two electrical images of the *rd10* RGC layer demonstrate that they occur at similar positions (**Fig. 6B**). The LFPs were restricted to areas of <200 µm in diameter. In the frequency band of 6 Hz the maximal power was ∼10^−4^ mV^2^/Hz (**Fig. 6C**), which is by a factor of 10 smaller than the maximal power in disinhibited, bleached retinas. It was also smaller than the power recorded in *rd1* retinas [7]. Importantly, only a small part of the measured retinal portion in *rd10* displayed rhythmic LFPs (**Fig. 6D**) and only in these areas rhythmic RGC activity was detected.

In summary, the low-frequency spatio-temporal dynamics differed in the four conditions. There were no detectable local field potentials in bleached retinas, weak LFPs in part of the *rd10* retinas, three - fold stronger LFPs in *rd1* retinas and even stronger static LFPs in disinhibited bleached *wt* retinas.

## Discussion

The experiments presented here indicate that (i) certain electrophysiological features of the *rd* phenotype can be obtained by partial bleaching a healthy retina but (ii) several physiological differences persist even after additional block of inhibitory receptors. Along the analysis of RGC spiking, of pair-wise spike train correlations and of LFP dynamics we compare in the following the physiology of healthy mouse retinas to the two *rd* phenotypes (rd1 and rd10) and discuss implications for vision restoration strategies.

### Functional properties of ganglion cells in bleached and dystrophic retinas

The augmented maintained and rhythmic activity in dystrophic mouse models has been established in many independent studies [3], [4], [5], [7] and confirmed here. In addition we have shown that an increase in maintained activity and induction of RGC rhythmicity can be generated in healthy retinas within hours using a relatively simple partial bleaching protocol. The RGC firing rates were elevated in all three conditions (partially bleached retina, *rd10* and *rd1*) as compared to control measurements. The values found here are higher than those reported in [4] but the qualitative trend is the same. The differences could be caused by different experimental conditions (i.e. ringer solution or light adaptation levels).

The elevated RGC firing rate is a strong indicator for an imbalance between excitatory and inhibitory driving force. As the firing rate is not changed in bleached retinas by additional blockade of inhibition, our data suggest an overall increase of excitation in bleached retinas. The short timescale in the bleaching experiments excludes any synaptic changes. An alternative explanation would be the increased excitability achieved by changes of the resting membrane potential of bipolar cells [10] or amacrine cells [11].

The smallest rhythmic fundamental frequency was detected in bleached retinas (4.3 Hz), the highest fundamental frequency was found in *rd1* (∼10 Hz). Several scenarios have been proposed to explain the different rhythmic frequencies in wild-type and dystrophic retinas. Yee et al. [8] hypothesize a slow rhythm generator in healthy retinas and multiple faster rhythm generators in dystrophic retinas. Our result may be in agreement with this hypothesis, however, in [8] the slow rhythm was revealed only upon blockade of inhibition. Under such conditions in our experiments (**Fig. 2**) the dystrophic *rd10* and the disinhibited retinas display the same fundamental frequency. We therefore favor a second scenario as proposed in [10], where oscillatory activity in *rd1* is explained by a change in the ion channel conductances of the electrically coupled ON cone bipolar – A2 amacrine network presynaptic to RGCs. Using blockers of the glutamergic excitatory RGC input the origin of this rhythmic activity is assigned here to presynaptic circuitry. We could not detect any remnant rhythmic RGC activity in bleached, in disinhibited or in *rd10* retinas after blocking the bipolar cell input. This result also excludes the hypothesis that gap-junctional coupling among RGCs or RGC pattern generators (i.e. melanopsin-containing RGCS) are responsible for the rhythmic RGC activity. The involvement of the AII amacrine cell in rhythm generation in partially bleached and rd10 retinas is supported by our finding of the fixed phase shift (∼70 msec) between neighbouring ON and OFF RGCs (separation distance <200 µm (**Fig. 3**) This result is in line with two recent studies in *rd1* retinas which report phase shifted-activity RGC activity [9] and oscillations of the trans-membrane voltage of *rd1* AII amacrine cells [11] Other presynaptic neurons have been reported to display oscillatory activity: starburst neurons [22], dopaminergic neurons [23], [24] as well as outer retinal neurons (W.Haq IOVS 2011: ARVO e-Abstract 1850).

The major retinal pathways, ON and OFF, are both affected in oscillating retinas. For the *rd10* retina we can provide only indirect evidence (**Fig. 3**), as around post-natal day 90 when the present recordings were performed, the RGCs do not respond to light. Remarkably, in bleached and probably in *rd10* retinas there were rhythmic RGCs of both polarities (ON and OFF) next to arrhythmic ones (**Fig. 3B**). This suggests that only certain retinal circuitries may undergo the above mentioned changes. The rhythmic activity recorded in many bleached OFF RGCs was somewhat unexpected since the loss of photoreceptor dark current should hyperpolarize and thus silence OFF bipolar cells. However, rhythmic activity in two types of OFF RGCs has been reported for adult *rd1* retinas [6]. More recently [8], [9], [11] suggested that in *rd1* oscillations in both RGC classes is driven by the same presynaptic rhythm generator. We speculate that the same rhythm generator may be acting in bleached retinas as well. This hypothesis is based on the similarity between phase-shifted activity between dissimilar RGC pairs [9]. The results presented in **Fig. 3F** **(**arbitrary phase shifted activity among *rd1* RGCs) seem to contradict the results presented by [9]; however in our recordings the RGC cell type is unknown. Taken together, our results suggest that in ‘disturbed’ retinas, there are strong interactions between the ON and OFF pathways questioning a clear separation between them. A more thorough analysis including RGC subtype clustering [25], [26] may reveal if all circuitries in dystrophic and bleached retinas are equally affected.

### Local field potentials in healthy and dystrophic retinas

Recent evidence indicates that active membrane currents and not synaptic current dominate the generation of LFPs [27]. We never identified local field potentials in bleached retinas indicating that there the circuitry presynaptic to each ganglion cell prevents strong correlated neuronal current in a large neuronal population. This is remarkable since a high percentage of RGCs displayed rhythmic activity (see also **Fig. 3B**). The emergence of LFPs in dystrophic and disinhibited retinas suggests that in these two cases a common mechanism could lead to correlated rhythmic activity in a large neuronal population. The emergence and propagation of local field potentials in disinhibited neural tissue is common to many other neural preparations such as the limbic system [28] or cortical areas [29].

Retinal local field potentials have been reported previously in dystrophic *rd1* and *rd10* retinas with fundamental frequencies in the same range as reported here [12], [21]. However, in previous studies the relatively large spacing between electrodes (200 µm) did not allow for a precise spatial characterization of these strong depolarisations. Here we identified clear differences between the propagating LFPs in *rd1* [*7*] and the LFPs in disinhibited C57Bl6 retinas as well as in unperturbed and disinhibited *rd10* (**Fig. 4** and **Fig. 5**). The propagation in *rd1* may be a residual of the developmental retinal waves which propagate across the ganglion cell layer until eye opening [30]. These propagating LFPs, which drive rhythmic RGC spiking, are responsible for the arbitrary phase shifts between rhythmic RGCs in these retinas (**Fig. 3F**). The rhythmic and localized LFPs in *rd10* retinas lead to constant phase shifts between rhythmic RGCs (**Fig. 3E**). Recently, propagating calcium waves have been reported in disinhibited adult mouse retinas [15]. Our results, obtained with a different recording technique and under slightly different experimental conditions, do not reveal propagating LFPs in disinhibited adult retinas. Future studies, combining the two methods in the same retina, are needed to resolve this open question.

### Implications for vision restoration strategies

Several approaches aimed to restore basic visual percept in blind patients assume a functional inner retinal circuitry. These approaches include electrical subretinal [31] or optogenetic approaches [32], [33], which utilizes the remaining retinal structures for signal conduction.

The functional similarity between bleached but otherwise healthy retinas and *rd10* retinas demonstrated here, suggests that in *rd10* the retinal circuitry may be less affected by photoreceptor loss. Further evidence is presented in [4], where the light response properties of wt and *rd10* RGCs at postnatal day 28 appear very similar, which is a strong indicator that basic circuitry evolved normally in *rd10*. Although in the *rd10* ganglion cell layer aberrant local field potentials were identified (**Fig. 6**) these were usually restricted to small areas. The *rd1* retina, on the other hand, seems to posses a more defective circuitry. Given the high degree of aberrant activity the application of the *rd1* model appears questionable [34]. However, we note that in the human degenerative disease of retinitis pigmentosa the degeneration of the PDE (as present in mouse rd) occurs very rare. Given that in humans RP is manifested after full circuitry development, we suggest considering *rd10* or a partially bleached and mildly disinhibited retina as a more appropriate model.

## Supporting Information

Figure S1 **Recovery of RGC light response after partial bleaching of a healthy retina.** (A) Light response of an OFF RGC to a full-field flicker stimulus prior to constant illumination recorded by one sensor of the CMOS MEA. The light onset is indicated above the recording trace. (B) Spontaneous activity of the same OFF RGC during constant illumination. (C) Activity recorded from the same cell to the light stimulus shown in (A) after the constant illumination. (D) Light response recorded from the same cell after continuous presentation of the flicker stimulus for several minutes.
(TIF)

## References

[1] Luo DG, Yau KW (2005) Rod sensitivity of neonatal mouse and rat. J. Gen. Physiol. 126: 263–269. 10.1085/jgp.200509342 PMC2266575 16129773

[2] Chang B, Hawes NL, Hurd RE, Davisson MT, Nusinowitz S, et al (2002) Retinal degeneration mutants in the mouse. Vis. Res. 42: 517–525. 10.1016/s0042-6989(01)00146-8 11853768

[3] Stasheff SF (2008) Emergence of sustained spontaneous hyperactivity and temporary preservation of OFF responses in ganglion cells of the retinal degeneration (rd1) mouse. J. Neurophys. 99: 1408–1421. 10.1152/jn.00144.2007 18216234

[4] Stasheff SF, Shankar M, Andrews MP (2011) Developmental time course distinguishes changes in spontaneous and light-evoked retinal ganglion cell activity in rd1 and rd10 mice. J. Neurophysiol. 105: 3002–3009. 10.1152/jn.00704.2010 21389300

[5] Borowska J, Trenholm S, Awatramani GB (2011) An Intrinsic Neural Oscillator in the Degenerating Mouse Retina. J. Neurosci. 31: 5000–5012. 10.1523/JNEUROSCI.5800-10.2011 PMC6622979 21451038

[6] Margolis DJ, Newkirk G, Euler T, Detwiler PB (2008) Functional stability of retinal ganglion cells after degeneration-induced changes in synaptic input. J (Neurosci.28) 6526–6536. 10.1523/JNEUROSCI.1533-08.2008 PMC3050548 18562624

[7] Menzler J, Zeck G (2011) Network oscillations in rod-degenerated mouse retinas. J. Neurosci. 31: 2280–2291. 10.1523/JNEUROSCI.4238-10.2011 PMC6633031 21307264

[8] Yee CW, Toychiev AH, Sagdullaev BT (2012) Network deficiency exacerbates impairment in a mouse model of retinal degeneration. Frontiers in systems neuroscience 6: 8. 22383900 10.3389/fnsys.2012.00008 PMC3285818

[9] Margolis DJ, Gartland AJ, Singer JH, Detwiler PB (2014) Network Oscillations Drive Correlated Spiking of ON and OFF Ganglion Cells in the rd1 Mouse Model of Retinal Degeneration. Plos One 9: e86253. 24489706 10.1371/journal.pone.0086253 PMC3904909

[10] Trenholm S, Borowska J, Zhang J, Hoggarth A, Johnson K, et al (2012) Intrinsic oscillatory activity arising within the electrically coupled AII amacrine-ON cone bipolar cell network is driven by voltage-gated Na plus channels. J. Physiol.London 590: 2501–2517. 22393249 10.1113/jphysiol.2011.225060 PMC3424767

[11] Choi H, Zhang L, Cembrowski MS, Sabottke CF, Markowitz AL, et al. (2014) Intrinsic bursting of AII amacrine cells underlies oscillations in the rd1 mouse retina. *in press* J Neurophysiol. (doi 10.1152/jn.00437.2014). 10.1152/jn.00437.2014 PMC4137253 25008417

[12] Goo YS, Ahn KN, Song YJ, Ahn SH, Han SK, et al (2011) Spontaneous Oscillatory Rhythm in Retinal Activities of Two Retinal Degeneration (rd1 and rd10) Mice. Korean Journal of Physiology & Pharmacology 15: 415–422. 22359480 10.4196/kjpp.2011.15.6.415 PMC3282230

[13] Soto F, Ma X, Cecil JL, Vo BQ, Culican SM, et al (2012) Spontaneous Activity Promotes Synapse Formation in a Cell-Type-Dependent Manner in the Developing Retina. Journal Of Neuroscience 32: 5426–5439. 22514306 10.1523/JNEUROSCI.0194-12.2012 PMC3353326

[14] Demas J, Sagdullaev BT, Green E, Jaubert-Miazza L, McCall MA, et al (2006) Failure to maintain eye-specific segregation in nob, a mutant with abnormally patterned retinal activity. Neuron 50: 247–259. 16630836 10.1016/j.neuron.2006.03.033

[15] Toychiev AH, Yee CW, Sagdullaev BT (2013) Correlated spontaneous activity persists in adult retina and is suppressed by inhibitory inputs. Plos One 8: e77658. 24204906 10.1371/journal.pone.0077658 PMC3812233

[16] Fain GL, Lisman JE (1993) Photoreceptor degeneration in vitamin-A deprivation and retinitis pigmentosa - the equivalent light hypothesis. Exp. Eye Res. 57: 335–340. 10.1006/exer.1993.1132 8224021

[17] Lambacher A, Vitzthum V, Zeitler R, Eickenscheidt M, Eversmann B, et al (2011) Identifying firing mammalian neurons in networks with high-resolution multi-transistor array (MTA). Applied Physics A 102: 1–11.

[18] Zeck G, Lambacher A, Fromherz P (2011) Axonal Transmission in the Retina Introduces a Small Dispersion of Relative Timing in the Ganglion Cell Population Response. Plos One 6: e20810. 21674067 10.1371/journal.pone.0020810 PMC3107248

[19] Petrusca D, Grivich MI, Sher A, Field GD, Gauthier JL, et al (2007) Identification and characterization of a Y-like primate retinal ganglion cell type. J Neurosci 27: 11019–11027. 17928443 10.1523/JNEUROSCI.2836-07.2007 PMC6672861

[20] Brivanlou IH, Warland DK, Meister M (1998) Mechanisms of concerted firing among retinal ganglion cells. Neuron 20: 527–539. 9539126 10.1016/s0896-6273(00)80992-7

[21] Ye JH, Goo YS (2007) The slow wave component of retinal activity in rd/rd mice recorded with a multi-electrode array. Physiological Measurement 28: 1079–1088. 17827655 10.1088/0967-3334/28/9/009

[22] Petit-Jacquea J, Volgyi B, Rudy B, Bloomfield S (2005) Spontaneous oscillatory activity of starburst amacrine cells in the mouse retina. Journal of Neurophysiology 94: 1770–1780. 15917322 10.1152/jn.00279.2005

[23] Atkinson CL, Feng J, Zhang D-Q (2013) Functional integrity and modification of retinal dopaminergic neurons in the rd1 mutant mouse: roles of melanopsin and GABA. Journal of Neurophysiology 109: 1589–1599. 23255724 10.1152/jn.00786.2012

[24] Feigenspan A, Gustincich S, Bean BP, Raviola E (1998) Spontaneous activity of solitary dopaminergic cells of the retina. Journal Of Neuroscience 18: 6776–6789. 9712649 10.1523/JNEUROSCI.18-17-06776.1998 PMC6792954

[25] Farrow K, Masland RH (2011) Physiological clustering of visual channels in the mouse retina. Journal of Neurophysiology 105: 1516–1530. 21273316 10.1152/jn.00331.2010 PMC3075295

[26] Zeck GM, Masland RH (2007) Spike train signatures of retinal ganglion cell types. Eur J Neurosci 26: 367–380. 17650112 10.1111/j.1460-9568.2007.05670.x

[27] Reimann M, Anastassiou C, Markram H, Koch C (2013) A biophysically detailed model of neocortical local field potentials predicts the critical role of active membrane currents. Neuron 79: 375–390. 23889937 10.1016/j.neuron.2013.05.023 PMC3732581

[28] Avoli M, de Curtis M (2011) GABAergic synchronization in the limbic system and its role in the generation of epileptiform activity. Progress in Neurobiology 95: 104–132. 21802488 10.1016/j.pneurobio.2011.07.003 PMC4878907

[29] Yang H, Shew WL, Roy R, Plenz D (2012) Maximal Variability of Phase Synchrony in Cortical Networks with Neuronal Avalanches. Journal Of Neuroscience 32: 1061–1072. 22262904 10.1523/JNEUROSCI.2771-11.2012 PMC3319677

[30] Blankenship AG, Feller MB (2011) Mechanisms underlying spontaneous patterned activity in developing neural circuits. Nature Reviews Neuroscience 11: 18–29. 10.1038/nrn2759 PMC2902252 19953103

[31] Zrenner E, Bartz-Schmidt KU, Benav H, Besch D, Bruckmann A, et al (2011) Subretinal electronic chips allow blind patients to read letters and combine them to words. Proceedings of the Royal Society B: Biological Sciences 278: 1489–1497. 21047851 10.1098/rspb.2010.1747 PMC3081743

[32] Busskamp V, Duebel J, Balya D, Fradot M, Viney TJ, et al (2010) Genetic Reactivation of Cone Photoreceptors Restores Visual Responses in Retinitis Pigmentosa. Science 329: 413–417. 20576849 10.1126/science.1190897

[33] Lagali PS, Balya D, Awatramani GB, Munch TA, Kim DS, et al (2008) Light-activated channels targeted to ON bipolar cells restore visual function in retinal degeneration. Nature Neuroscience 11: 667–675. 18432197 10.1038/nn.2117

[34] Cameron MA, Suaning GJ, Lovell NH, Morley JW (2013) Electrical Stimulation of Inner Retinal Neurons in Wild-Type and Retinally Degenerate (rd/rd) Mice. Plos One 8: e68882. 23874798 10.1371/journal.pone.0068882 PMC3708954

